# Effectiveness of intra-alveolar chlorhexidine gel in reducing dry socket following surgical extraction of lower third molars. A pilot study

**DOI:** 10.4317/jced.52444

**Published:** 2016-04-01

**Authors:** Silvana Requena-Calla, Italo Funes-Rumiche

**Affiliations:** 1 Dentist Surgeon; 2 Dentist Surgeon, Specialist in Oral and Maxillofacial Surgery

## Abstract

**Background:**

Dry socket is one of the most studied complications in dentistry and several studies have sought an effective and safe method for its prevention and treatment. The aim of this study was to evaluate the effectiveness of intra-alveolar gel chlorhexidine in preventing dry socket after the surgical removal of third molars.

**Material and Methods:**

The sample involved the treatment of 40 patients who required extraction of third molars impacted, which were randomly assigned to research groups: experimental group (chlorhexidine gel 0.12%) and control group (placebo gel). Performed the extraction was administered 1 mL of chlorhexidine gel or 1 mL of placebo gel within the socket. The removal of suture was on the fifth postoperative day in which the presence or absence of dry socket was evaluated.

**Results:**

No relationship between the appearance of dry socket after application of chlorhexidine gel or placebo gel (X2 test, *p* = 0.311) was found. However, significant differences (U Mann-Whitney test, *p* = 0.036) in the pain presented on the fifth postoperative day were evident (VAS).

**Conclusions:**

The administration of intra-alveolar chlorhexidine gel 0.12% could generate a better response to postoperative pain after the removal of third molars.

** Key words:**Third molar surgery, dry socket, chorhexidine gel.

## Introduction

Dry socket is the most common post-operative complication after tooth extraction. Its frequency varies between 1 and 4% of all tooth extractions, and can reach 20 to 30% in third molar surgeries, temporarily reducing the quality of life of patients because that causes a sharp pain (1). The etiology of this condiction is not clearly known, prevention remains the main therapeutic weapon that is available, there have been significant efforts to achieve an adequate and effective protocol aimed at reducing the prevalence of the disease, particularly since the clinical view (2).

This condition can be defined as a postoperative pain in the socket and around it, which increases between the first and third postoperative day, accompanied by a total or partial disintegration of the clot intraalveolar with or without the presence of halitosis, which temporarily reduces the quality of life of patients (3).

Within antiseptics, chlorhexidine has proved a good prophylactic agent for dry socket (4-7).

Chlorhexidine presenting as gel is matter for investigation. The drug can be placed within the alveolus, enabling a more direct action on the socket; and gel form allows more prolonged action in time, compared with chlorhexidine rinse (8-10).

## Material and Methods

The present research is a randomized, double-blind study. A placebo gel, containing only excipients in its composition, was ordered to develop to Laboratory of Pharmacy and Biochemistry of Universidad Nacional Mayor de San Marcos (Peru).

This study was done on 40 patients who were treated at the Department of Oral Surgery Maxillofacial of the Naval Medical Center “Santiago Tavara” from April to August 2014, which required the removal of impacted third molars. They were included in the study patients with ASA I classification, both genders, between 16 and 40 years. Exclusion criteria were patients with immuno-depression, AIDS, pregnancy or women in the lactating period, smokers, patients taking oral contraceptives, patients in the use of epinephrine is contraindicated and allergics to NSAIDs.

The research was approved by the Ethics Committee of the Naval Medical Center “Santiago Tavara”. All patients who agreed to participate voluntarily signed an informed consent.

Patients were randomly assigned to research groups: experimental group (n = 20), control group (n = 20).

Surgical extraction of third molars were performed, surgery time was measured from the start of the incision until the avulsion of the tooth. This study used the New Gbotolorum index to quantify the degree of surgical difficulty (11). To standardize the amount of research products, 1 ml of chlorhexidine gel or placebo gel 1ml was administered into the socket. Black silk suture 4/0 for wound closure was used by secondary intention. The indications and pharmacological treatments were the same for both groups; 200 mg of celecoxib, 500 mg of paracetamol and 500 mg of amoxicillin. Some patients needed to add parenteral medication (100 mg ketoprofen and 4 mg dexamethasone). The removal of the sutures was performed at 5 days post-operative day in which the presence or absence of alveolitis was evaluated.

To analyze and determine the differences between the two groups (control and experimental) Chi square test (X2) was used for comparison of qualitative variables. U Mann-Whitney test was used for comparison of quantitative variables.

## Results

A total of 17 women and 23 men were treated. A total of 40 lower impacted third molars were operated (24 left and 16 right). The average age was 22.98 ± 6.318 years. The average effective time surgery was 8.55 ± 6.116 min. The average degree of surgical difficulty was 7.58 ± 1.217 according to Gbotolorum index.

In relation to the incidence of dry socket, we found a single case of the patology in the control group and no cases were observed in the experimental group. No relationship between the incidence of dry socket and the type of intra-alveolar gel administration was found (Chi square test, *p* = 0.311).

The pain experienced by patients was significantly lower in the experimental group compared with the control on the fifth postoperative day (*p* = 0.036, U Mann-Whitney test) ( Table 1).

**Table 1:**
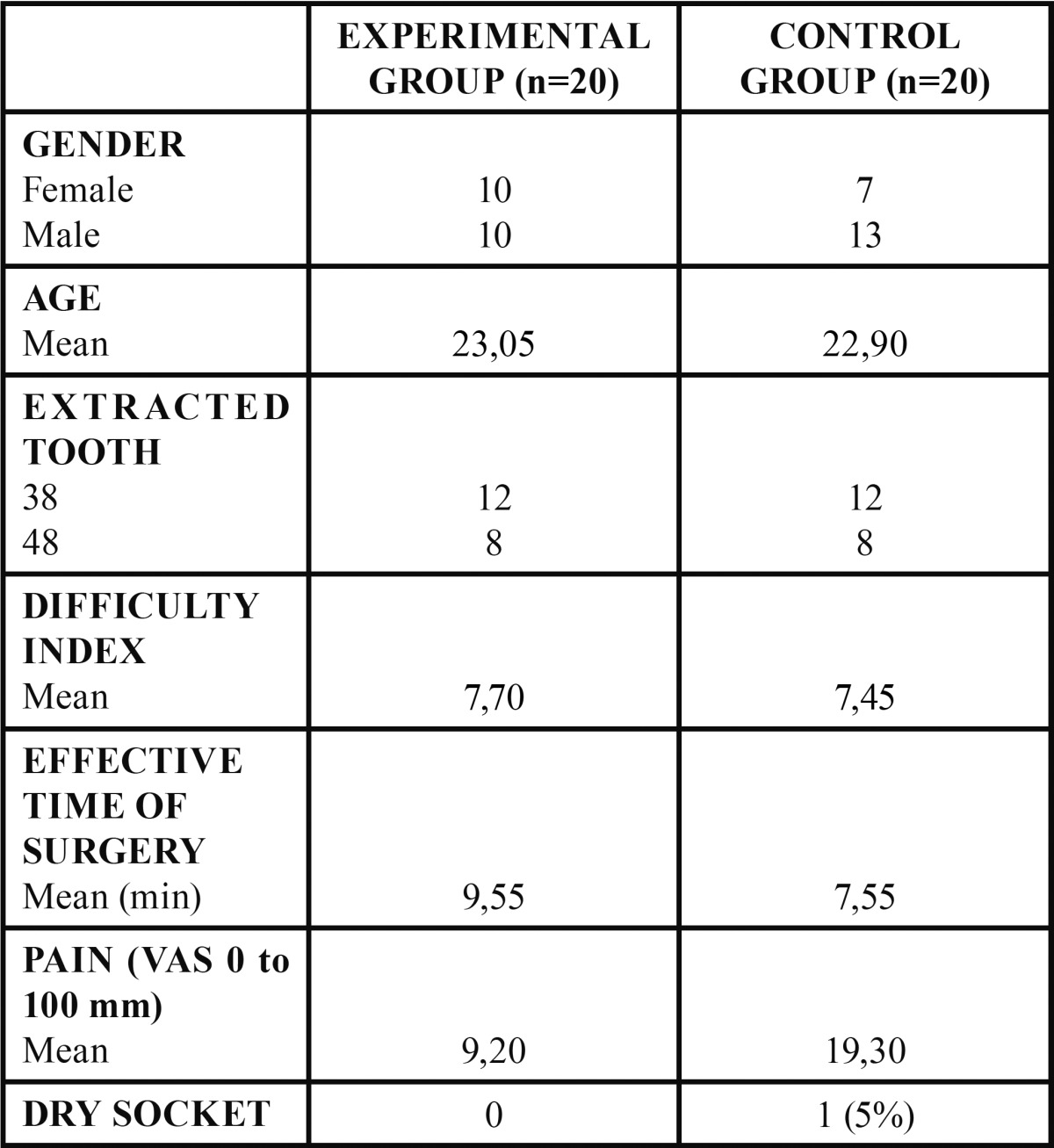
Description and comparison of patients.

## Discussion

Dry socket is one of the most common postoperative complications, and the incidence increases after extraction of third molars. The clinical of this disease is characteristic: the presence of severe pain in surgical area from the third postoperative day by the presence of naked alveolus caused by the breakdown of blood clots; resulting in decreased quality of life of patients (12).

Blum (2002) published a systematic review of dry socket, which states that the disintegration of the blood clot by increased fibrinolytic activity due to the presence of oral microorganisms, the difficulty and length of the surgical trauma are possible causes most studied and mentioned by other authors (3).

The incidence of dry socket varies from 1-4% after routine dental extractions, but the values increase to 45% after lower third molars surgeries (3,12).

Haraji *et al.* (2012) found a high prevalence of the disease, 27.5% of dry socket in patients undergoing impacted third molar surgery (13). Torres Lagares *et al.* (2006) reported 20% of dry socket in patients who underwent impacted third molar surgery (8). Similar results were obtained by Hita Iglesias *et al.* (2008) (9) and Tek *et al.* (2014) (14) about the prevalence of dry socket, where 15% of patients develop dry socket after the extraction of impacted third molars. However, Rodriguez Perez *et al.* (2013) reported only 10% of incidence of dry socket in patients who underwent mandibular third molar surgery (15). In this study the incidence of dry socket after surgical treatment of third molars was very low (2.5% of all samples; 5% in the control group) compared with previous studies that demonstrated an increase in the occurrence of pathology after third molar surgery. In concordance with us Sridhar *et al.* (2011) observed 4% of incidence of dry socket in total sample (8% in the control group) after third molar impacted extraction (16).

Perhaps the main reason for the low incidence of alveolitis were aseptic and antiseptic measures taken by health personnel, scheduled surgeries were performed in the operating room of the hospital center with strict biosecurity measures and surgical instruments and other materials were autoclaved.

In addition to the above, must take into account the experience of the surgeon, who proved a short effective surgery time (mean = 8.55 min). Similarly, Santana *et al.* (2013) found better results in terms of trismus, inflammation, mouth opening and postoperative pain in patients who underwent impacted third molar surgery with an operating time between 0-15 min (17). Also, in the study by Eshghpour *et al.* (2013) was evident a decrease in the incidence of dry socket in the group of patients who underwent impacted third molar surgery with lower or equal surgical time to 8.24 min (18). According the skill and experience of the specialist, Oginni *et al.* (2003) reported that the occurrence of dry socket was higher in patients who were treated by undergraduates, agreeing with studies that have identified the skill of the surgeon as a risk factor in the development of dry socket (19).

As for the extraction third molar difficulty, Haraji *et al.* (2014) found a positive association between increase difficulty of third molar surgery and the risk of dry socket (20). However, in this research the average of surgical difficulty in both study groups was moderate (as Gbotolorum index), which would result in the increase of dry socket. In contradiction to this and despite the moderate degree of surgical difficulty, a high incidence of dry socket was not obtained.

Regarding measures to prevent the occurrence of dry socket, Blum (2002) emphasized the use of antiseptic agents, where chlorhexidine rinse produces reduction in the incidence of dry socket after third molar surgery (3).

Torres Lagares *et al.* (2006) found significant differences (*p* = 0.019) between the incidence of dry socket and chlorhexidine gel intra-alveolar administration (8). Hita Iglesias *et al.* (2008) found a statistically significant decrease of 70% in the incidence of dry socket in the intra-alveolar chlorhexidine gel group compared to the group where was administered chlorhexidine rinse (*p*= 0.040) (9). In this study, no relationship was found as to the decrease of dry socket and 0.12% chlorhexidine gel intraalveolar administration. These results are similar to found by Torres Lagares *et al.* (2006), where no significant difference was evident in the incidence of dry socket and chlorhexidine gel intra-alveolar administration (21).

Concerning to perception of postoperative pain, authors like Haraji *et al.* (2014) found a positive correlation between the levels of perceived pain on the first and third postoperative day, where participants of the experimental group (intra-alveolar 0.2 % chlorhexidine gel) reported better response to postoperative pain compared to the placebo group (22). In this study were found statistically significant differences between the study groups for postoperative pain (*p* = 0.036), but the measure was only carried out on the fifth postoperative day considering that the disease has an appearance between the first and third day after the tooth extraction and 95-100% of dry socket’s cases reported in the literature were expressed in postoperative week (Blum, 2002) (3). Other authors such as Rodríguez Pérez *et al.* (2013) (15) and Torres Lagares (2006) (21) reported no significant differences in pain presented by the patients during the first postoperative week.

No previous studies examining the efficacy of chlorhexidine gel as postoperative analgesic. However, it can be assumed that the antiseptic quality chlorhexidine gel reduces the microbial population in the surgical site and thus inflammatory mediators that are produced as a result of bacterial activity, thereby the painful inflammatory response is reduced.

Given the sample size of our study, data presented they must be taken with caution and corroborated by subsequent studies that use a larger sample size.
